# Variation in short-term and long-term responses of photosynthesis and isoprenoid-mediated photoprotection to soil water availability in four Douglas-fir provenances

**DOI:** 10.1038/srep40145

**Published:** 2017-01-10

**Authors:** Laura Verena Junker, Anita Kleiber, Kirstin Jansen, Henning Wildhagen, Moritz Hess, Zachary Kayler, Bernd Kammerer, Jörg-Peter Schnitzler, Jürgen Kreuzwieser, Arthur Gessler, Ingo Ensminger

**Affiliations:** 1 Department of Biology, Graduate Programs in Cell & Systems Biology and Ecology & Evolutionary Biology, University of Toronto, 3359 Mississauga Road, Mississauga, ON, Canada; 2 Forstliche Versuchs- und Forschungsanstalt Baden-Württemberg, Wonnhaldestr. 4, 79100 Freiburg, Germany; 3 Institute of Bio and Geosciences IBG-2, Plant Sciences, Forschungszentrum Jülich GmbH, Jülich, Germany; 4 Chair of Tree Physiology, Institute of Forest Sciences, Albert-Ludwigs University Freiburg, Georges-Köhler Allee 53, 79110 Freiburg, Germany; 5 Institute for Landscape Biogeochemistry, Leibniz Centre for Agricultural Landscape Research (ZALF), Eberswalderstr. 84, 15374 Müncheberg, Germany; 6 Institute of Ecology, Leuphana University of Lüneburg, Scharnhorststr. 1, 21335 Lüneburg, Germany; 7 HAWK University of Applied Sciences and Arts Hildesheim/Holzminden/Göttingen, Faculty of Resource Management, Büsgenweg 1A, 37077 Göttingen, Germany; 8 Institute of Biology III, Faculty of Biology, Albert-Ludwigs University Freiburg, Schänzlestr. 1, 79104 Freiburg, Germany; 9 USDA Forest Service, Northern Research Station, Lawrence Livermore National Laboratory, Livermore, California 94550, United States of America; 10 Centre for Biosystems Analysis (ZBSA), Albert-Ludwigs-University Freiburg, Habsburgerstr. 49, 79104 Freiburg, Germany; 11 Research Unit Environmental Simulation, Institute of Biochemical Plant Pathology, Helmholtz Zentrum München, Ingolstädter Landstr. 1, 85764 Neuherberg, Germany; 12 Berlin-Brandenburg Institute of Advanced Biodiversity Research (BBIB), 14195 Berlin, Germany; 13 Swiss Federal Research Institute WSL, Zürcherstr. 111, 8903 Birmensdorf, Switzerland

## Abstract

For long-lived forest tree species, the understanding of intraspecific variation among populations and their response to water availability can reveal their ability to cope with and adapt to climate change. Dissipation of excess excitation energy, mediated by photoprotective isoprenoids, is an important defense mechanism against drought and high light when photosynthesis is hampered. We used 50-year-old Douglas-fir trees of four provenances at two common garden experiments to characterize provenance-specific variation in photosynthesis and photoprotective mechanisms mediated by essential and non-essential isoprenoids in response to soil water availability and solar radiation. All provenances revealed uniform photoprotective responses to high solar radiation, including increased de-epoxidation of photoprotective xanthophyll cycle pigments and enhanced emission of volatile monoterpenes. In contrast, we observed differences between provenances in response to drought, where provenances sustaining higher CO_2_ assimilation rates also revealed increased water-use efficiency, carotenoid-chlorophyll ratios, pools of xanthophyll cycle pigments, β-carotene and stored monoterpenes. Our results demonstrate that local adaptation to contrasting habitats affected chlorophyll-carotenoid ratios, pool sizes of photoprotective xanthophylls, β-carotene, and stored volatile isoprenoids. We conclude that intraspecific variation in isoprenoid-mediated photoprotective mechanisms contributes to the adaptive potential of Douglas-fir provenances to climate change.

The response of plant species to limited water availability varies widely, but there is also considerable variation within species and among populations^1^,^2^. With increases in drought and heat globally as a consequence of climate change, the survival of species may become dependent on populations that are inherently pre-adapted to drier and hotter climate^3^,^4^. For long-lived forest tree species, intraspecific variation in response to water availability contributes to their ability to cope with and adapt to climate change. Douglas-fir (*Pseudotsuga menziesii*) is one of the most ecologically and economically important tree species in Europe and in its origin, the Western USA and Canada^5^. Douglas-fir thrives under diverse climatic conditions and thus has a wide distribution^6^. The coastal subspecies (*var. menziesii*) which originates from the humid maritime climate along the West Coast typically shows higher wood productivity compared to the interior subspecies (*var. glauca*), which occurs under dry conditions in the continental mountain regions^6^. Provenances of both varieties show high genetic and phenotypic diversity^7^ and vary in their drought tolerance and productivity under drought^8^. Provenances that originate from dry environments are known to cope better with water limitations and drought stress conditions, but little is known about the physiological mechanisms contributing to drought tolerance^4^.

Conifers are generally well drought-adapted and exhibit a conservative regulation of stomatal conductance (g_s_) to minimize water loss^9^. Intraspecific variation in morphological traits, such as a root-shoot ratio and xylem resistance to cavitation, as well as physiological traits, such as the efficiency of the regulation of g_s_ and intrinsic water use efficiency (IWUE) contributes to provenances’ drought tolerance and allows to maintain higher assimilation rates (A) under drought conditions. Nevertheless, reduced A under drought conditions leads to the formation of reactive oxygen species (ROS), which impose photooxidative stress^10^. Therefore, drought enhances the demand for photoprotective mechanisms such as non-photochemical quenching (NPQ), scavenging of ROS, or production and emission of volatiles. These mechanisms are often mediated by isoprenoids^11^,^12^.

Essential isoprenoids include the photosynthetic pigments, chlorophylls and carotenoids^13^. The pigment composition of the photosynthetic apparatus reflects long-term adjustments in response to environmental conditions and varies across species due to adaptation to different environments^14^,^15^,^16^,^17^. The xanthophyll cycle pigment pool size in relation to chlorophylls determines the photoprotective capacity of a plant and is increased in response to drought in many plant species^13^, e.g. in species of the genus *Quercus*^18^. In contrast to these long-term adjustments, the de-epoxidation of the xanthophyll cycle pigments in response to excess energy provides an instantaneous mechanism to quench excess light energy and facilitate NPQ^10^,^19^. In addition to the well-known xanthophyll cycle, β-carotene protects the photosystem reaction centers by chemical scavenging of ROS and undergoes oxidation by an electron transport reaction^20^,^21^. The breakdown of β-carotene results in metabolites that are involved in plant signalling^21^,^22^. In line with this finding, β-carotene has the highest turnover rates among the major carotenoids in *Arabidopsis*, indicating a constant replenishment of β-carotene pool sizes^23^. β-carotene per chlorophylls can increase in response to irradiance, but the adjustments of β-carotene pool sizes under drought stress are unknown^13^.

In addition to essential isoprenoids, many tree species including Douglas-fir produce non-essential volatile isoprenoids^24^. Pools of stored volatile monoterpenes and sesquiterpenes provide a source for emission in response to biotic and abiotic stress and are adjusted to prevailing environmental conditions^25^. The emission of non-essential isoprenoids is a rapid response mechanism to protect plants from thermal and oxidative damage^26^. The biosynthesis of non-essential isoprenoids also serves as an important metabolic sink for electrons that result from the uptake of excess energy^27^. However, emission of volatile isoprenoids can also contribute to a significant loss of previously fixed carbon. Transgenic tobacco plants emitting isoprene showed lower ROS levels and lipid oxidation compared to non-isoprene-emitting tobacco, but also exhibited reduced growth^28^. In beech seedlings, monoterpene emissions resulted in decreased growth when seedlings were exposed to drought^29^.

Differences in short- and long-term responses of isoprenoid metabolism might contribute to intraspecific variation in photosynthetic carbon assimilation and photoprotective mechanisms under drought. Variation in the response of photosynthetic gas exchange from different Douglas-fir provenances is indicated by provenance-specific variation in growth performance (height and radial growth) in response to water-limiting conditions^8^,^30^, provenance-specific variation in carbon isotope composition^30^,^31^ and differences in provenance susceptibility to xylem cavitation^32^. Consequently, Douglas-fir provides an ideal model to study provenance-specific variation of the isoprenoid-mediated photoprotective mechanisms.

The aim of this study was to assess which isoprenoid-mediated photoprotective mechanisms are induced in response to drought stress in different Douglas-fir provenances. We hypothesized that all provenances show short-term responses such as enhanced NPQ, increased xanthophyll cycle de-epoxidation state and emission of non-essential volatile isoprenoids. Furthermore, provenances that exhibit lower assimilation rates are expected to show pronounced long-term adjustments compared to provenances which are able to maintain higher assimilation rates, including increased pools of xanthophyll cycle pigments and stored volatile isoprenoids.

The short- and long-term adjustments of photosynthesis, photosynthetic pigments as well as emission and pool sizes of monoterpenes in response to soil water availability were studied in 50-year-old trees of four Douglas-fir provenances grown in two provenance trials in south-western Germany.

## Results

### Environmental conditions

**Figure 1:**
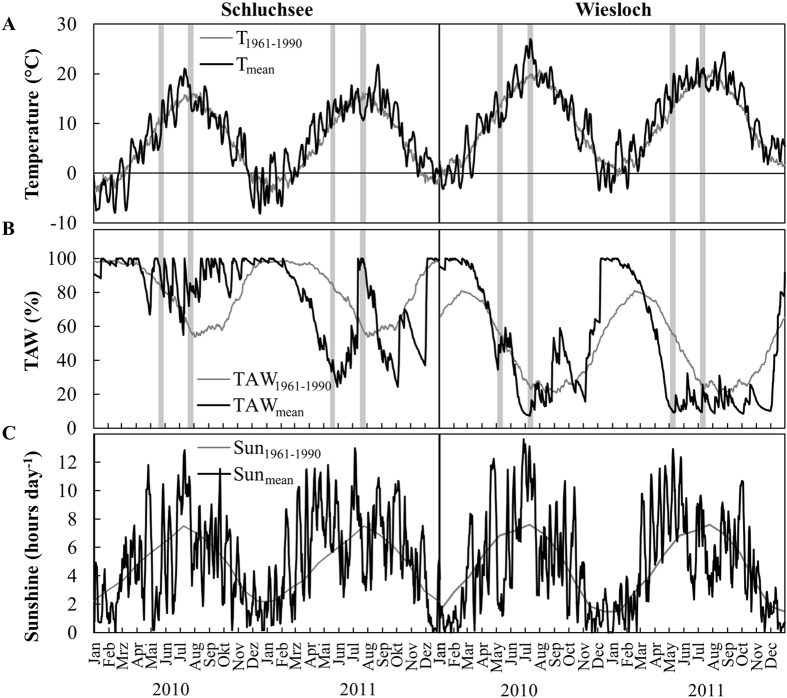
Annual climate at the field sites in 2010 and 2011 and for a 30-year reference period (1961–1990). (**A**) 5-day running average of mean daily temperature (T_mean_) and mean daily temperature for the reference period (T_1961–1990_), (**B**) mean daily total available soil water for 2010 and 2011 (TAW_mean_) and the reference period (_T1961–1990_), and (**C**) sunshine duration in hours per day in 2010 and 2011 (Sun_mean_) and the reference period (Sun_1961–1990_) at the two field sites Schluchsee and Wiesloch. Gray bars indicate time of the 8 measurement campaigns.

The field site Wiesloch was generally warmer, drier and sunnier than Schluchsee, indicated by higher temperatures, lower total available soil water (TAW), and higher sunshine duration per day (Fig. 1A–C). Differences in TAW and precipitation were reflected in lower pre-dawn and midday water potential at the drier site Wiesloch compared to Schluchsee (Table 1). We also observed inter-annual differences, with approximately 2 °C higher mean temperature and 30% lower mean precipitation in 2011 compared to 2010 at both field sites. Warmer and drier spring conditions in 2011 resulted in an earlier onset of the growing season, indicated by an earlier budburst in 2011 (Supplementary Table S1).

### Site-and provenance-specific variation in photosynthesis and isoprenoid metabolism

**Figure 2:**
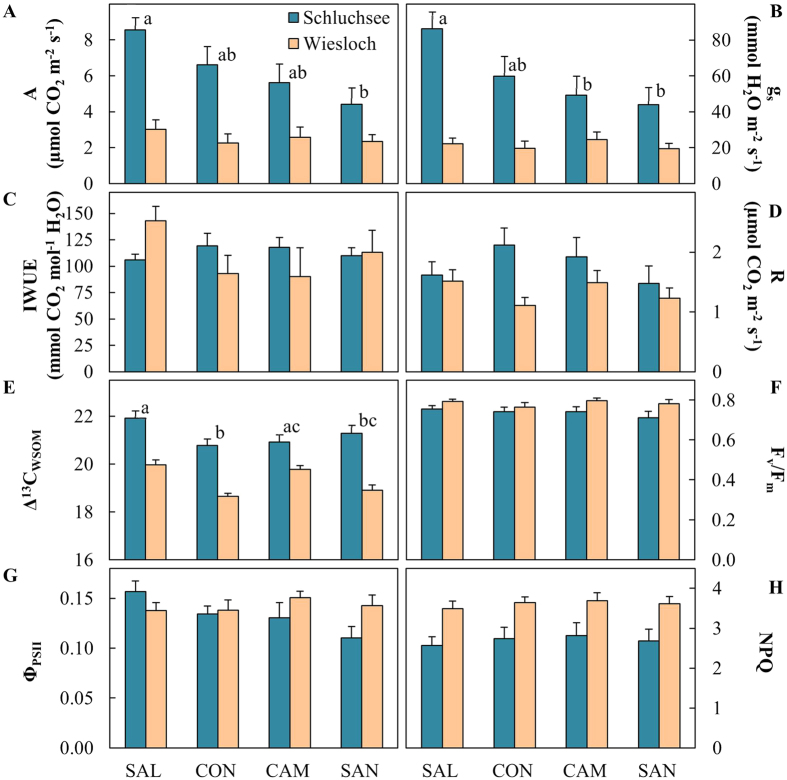
Photosynthesis parameters of four Douglas-fir provenances averaged over May and July of 2010 and 2011 for two field sites, Schluchsee and Wiesloch. (**A**) Assimilation rates (**A**), (**B**) stomatal conductance (g_s_), (**C**) intrinsic water-use efficiency (IWUE), (**D**) dark respiration rate (R), (**E**) carbon stable isotope discrimination (Δ^13^C_WSOM_), (**F**) maximum quantum yield of dark-adapted needles (F_v_/F_m_), (**G**) effective quantum yield of light-adapted needles (Φ_PSII_), and (**H**) non-photochemical quenching (NPQ). Data obtained from Schluchsee in May 2010 was excluded (see material and methods for further details). Significant differences between provenances (p < 0.05) are indicated by different letters.

The comparison of four Douglas-fir provenances at two field sites revealed provenance- and site-specific variation of photosynthetic gas exchange and chlorophyll fluorescence (Fig. 2, Table 2). Net CO_2_ assimilation rates (A) and stomatal conductance (g_s_) were generally higher in Schluchsee than in Wiesloch, and showed provenance-specific variation (Fig. 2A,B, Table 2). All provenances showed low A and g_s_ in Wiesloch, but the Salmon Arm (SAL) provenance showed significantly higher A and g_s_ compared to Santiam River (SAN), with intermediate rates for Conrad Creek (CON) and Cameron Lake (CAM), at the Schluchsee site. In contrast, intrinsic water-use efficiency (IWUE, measured as A/g_s_) and dark respiration (R) were variable and showed no consistent difference between sites and provenances (Fig. 2C,D, Table 2). As a proxy that integrates IWUE over several days we also estimated discrimination against stable carbon isotopes in water-soluble organic matter of needles (Δ^13^C_WSOM_). Low Δ^13^C_WSOM_ in Wiesloch compared to Schluchsee demonstrated significantly increased IWUE, and revealed considerable variation among provenances (Fig. 2E). Overall, CON showed the lowest Δ^13^C_WSOM_ values and thus highest IWUE, compared to highest Δ^13^C_WSOM_ in SAL and intermediate values for CAM and SAN (Fig. 2E, Table 2).

Maximum quantum yield of dark-adapted needles (F_v_/F_m_) was generally higher at Wiesloch compared to Schluchsee, but did not exhibit differences among provenances (Fig. 2F, Table 2). The effective quantum yield (Φ_PSII_) of light-adapted needles revealed minor differences between provenances at the field site Schluchsee, with higher values in SAL compared to the coastal provenances (Fig. 2G, Table 2). Non-photochemical quenching (NPQ), an indicator of photoprotective quenching of excess light energy, was higher in Wiesloch compared to Schluchsee, but the differences between provenances were not significant (Fig. 2H, Table 2).

**Figure 3:**
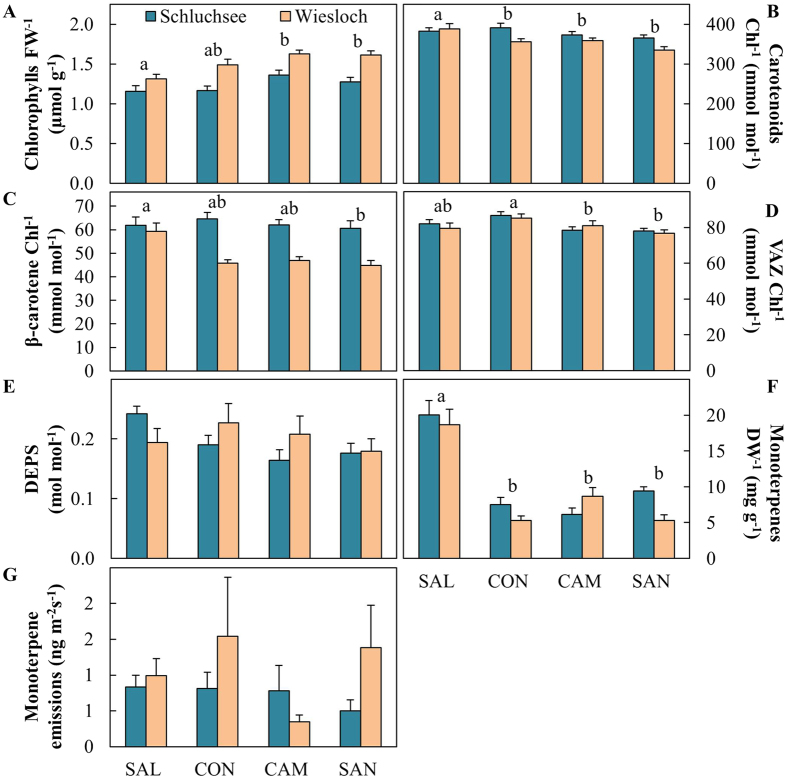
Photosynthetic pigments and volatile isoprenoid parameters averaged over May and July of 2010 and 2011 for two field sites, Schluchsee and Wiesloch. (**A**) total chlorophyll a and b per fresh weight (chlorophylls FW^−1^), (**B**) carotenoids per total chlorophyll (Carotenoids Chl^−1^), (**C**) β-carotene per total chlorophyll (β-carotene Chl^−1^), (**D**) xanthophyll cycle pigments per total chlorophyll (VAZ Chl^−1^), (**E**) de-epoxidation status of the xanthophyll cycle pigments (DEPS), (**F**) total stored monoterpenes per dry weight (monoterpenes), and (**G**) total monoterpene emissions (emitted monoterpenes). Data obtained from Schluchsee in May 2010 was excluded (see material and methods for further details). Significant differences between provenances (p < 0.05) are indicated by different letters.

Chlorophyll content was higher in Wiesloch compared to Schluchsee and varied between provenances (Fig. 3A, Table 2). SAL showed the lowest chlorophyll content, CON was intermediate and CAM and SAN showed the highest content. Similarly, carotenoids per chlorophyll were influenced by site and provenance (Table 2). Carotenoid content was generally higher in Schluchsee (Fig. 3B), and significantly higher in SAL compared to the coastal provenances CON, CAM and SAN. β-carotene was highest in SAL, and lowest in SAN and intermediate in CON and CAM (Fig. 3C, Table 2). The pool of xanthophyll cycle pigments (VAZ) varied significantly between provenances, with highest VAZ-pools in CON, intermediate pools in SAL and lowest pools in CAM and SAN at both field sites (Fig. 3D, Table 2). The de-epoxidation state of the VAZ-pool (DEPS) did not vary among provenances and sites (Fig. 3E, Table 2).

The needle concentrations of stored monoterpenes varied slightly among sites, but the overall differences between provenances with about three times higher monoterpenes in the interior provenance SAL than in any of the coastal provenances exceeded site-specific differences by far (Fig. 3F, Table 2). Monoterpene emission rates did not significantly vary between provenances and field sites (Fig. 3G, Table 2).

### Variation in photosynthesis and isoprenoid metabolism in response to environmental factors

As a second step of analysis, we aimed to illustrate the response of Douglas-fir to changes in environmental conditions and assessed the correlation of each physiological parameter with the three environmental factors total available soil water (*TAW*), sunshine duration on the day of measurement (*Sun*), and mean temperature of the day of measurement (*Temperature*). In order to assess the response of Douglas-fir to changes in environmental conditions, physiological parameters were plotted against the environmental factor that showed the highest correlation.

**Figure 4:**
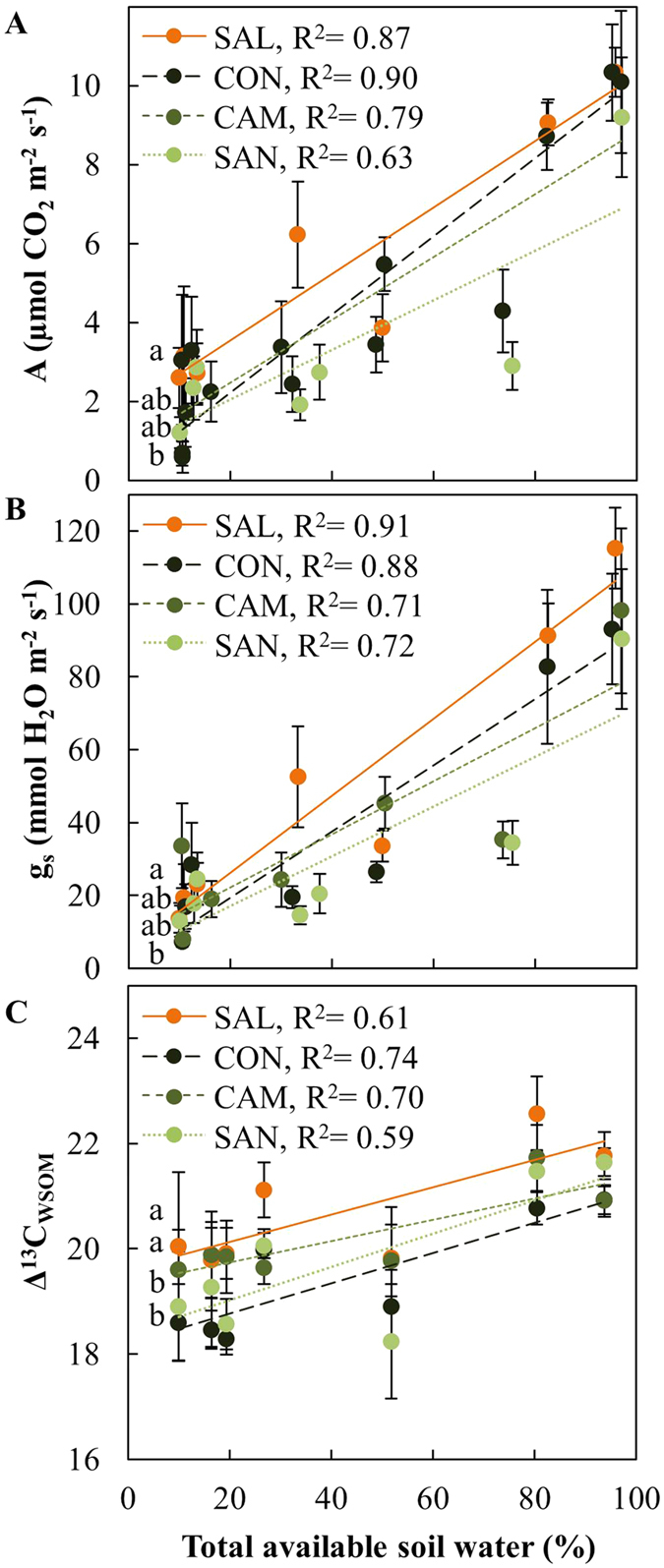
Photosynthetic gas exchange parameters of four Douglas-fir provenances in response to total available soil water. (**A**) assimilation rates (**A**), (**B**) stomatal conductance (g_s_), and (**C**) carbon stable isotope discrimination (Δ^13^C_WSOM_) were measured in May and July of 2010 and 2011 at two field sites, Schluchsee and Wiesloch. Data for A and g_s_ show means of n = 5–6 measurements (±SE) at a light intensity of 1000 μmol m^−2^ s^−1^, data for Δ^13^C_WSOM_ was obtained from n = 5–6 samples of previous year needles (±SE). Data obtained from Schluchsee in May 2010 was excluded (see material and methods for further details). Significant differences between provenances (p < 0.05) are indicated by different letters.

For A and g_s_, we observed a strong limitation of all provenances by low TAW (Fig. 4A,B). Maximum rates of photosynthetic gas exchange were observed in all provenances when TAW exceeded 90% in July 2011 in Schluchsee (Supplementary Fig. S1A,B). When TAW was below 20% as well as in May 2010, when low temperature conditions were prevailing, A and g_s_ were low in all provenances (Fig. 4A,B, Supplementary Fig. S1a). All provenances showed a decrease in A and g_s_ in response to decreased TAW, but varied significantly in their response to environmental conditions, as indicated by significant genotype by environment interaction (Table 2). Especially when TAW was low, Salmon Arm (SAL) showed significantly higher rates compared to Santiam River (SAN), with intermediate rates for Conrad Creek (CON) and Cameron Lake (CAM) (Fig. 4A,B).

Δ^13^C_WSOM_ decreased in all provenances in response to decreases in TAW, demonstrating increased IWUE when TAW was low (Fig. 4C). Δ^13^C_WSOM_ was significantly lower in CON and SAN, indicating higher IWUE compared to SAL and CAM. Such provenance-specific differences were especially apparent in July, e.g. when the highest Δ^13^C_WSOM_ in SAL trees occurred in July 2010 in Schluchsee, or in SAL and CAM trees in July 2010 and July 2011 in Wiesloch (Supplementary Fig. S1D).

**Figure 5:**
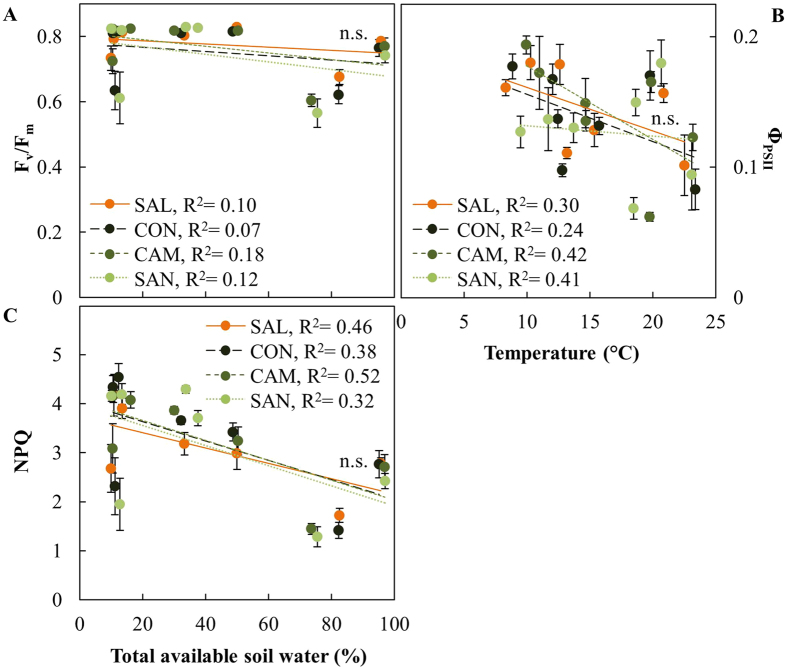
Chlorophyll fluorescence parameters of four Douglas-fir provenances in response to total available soil water and temperature, respectively. (**A**) maximum quantum yield of dark-adapted needles (F_v_/F_m_), (**B**) effective quantum yield of light-adapted needles (Φ_PSII_), and (**C**) non-photochemical quenching (NPQ) were measured in May and July of 2010 and 2011 at two field sites, Schluchsee and Wiesloch. Data show means of n = 5–6 measurements (±SE) at a light intensity of 1000 μmol m^−2^ s^−1^. Data obtained from Schluchsee in May 2010 was excluded (see material and methods for further details). Significant differences between provenances (p < 0.05) are indicated by different letters.

F_v_/F_m_ was generally around 0.8 and only decreased during the exceptionally warm and dry July 2010 (Supplementary Fig. S2A). F_v_/F_m_ was slightly lower at the moist site Schluchsee, which contributed to a negative relationship between TAW and F_v_/F_m_ (Fig. 5A). Φ_PSII_ was the only parameter that correlated best with temperature, with lower values as temperature increased (Fig. 5B), and a minimum under hot conditions in July 2010 (Supplementary Fig. S2B). NPQ increased in response to low TAW in all provenances (Fig. 5C). High NPQ also occurred under low temperature conditions in May 2010 at the beginning of the growing season (Supplementary Fig. S2C).

**Figure 6:**
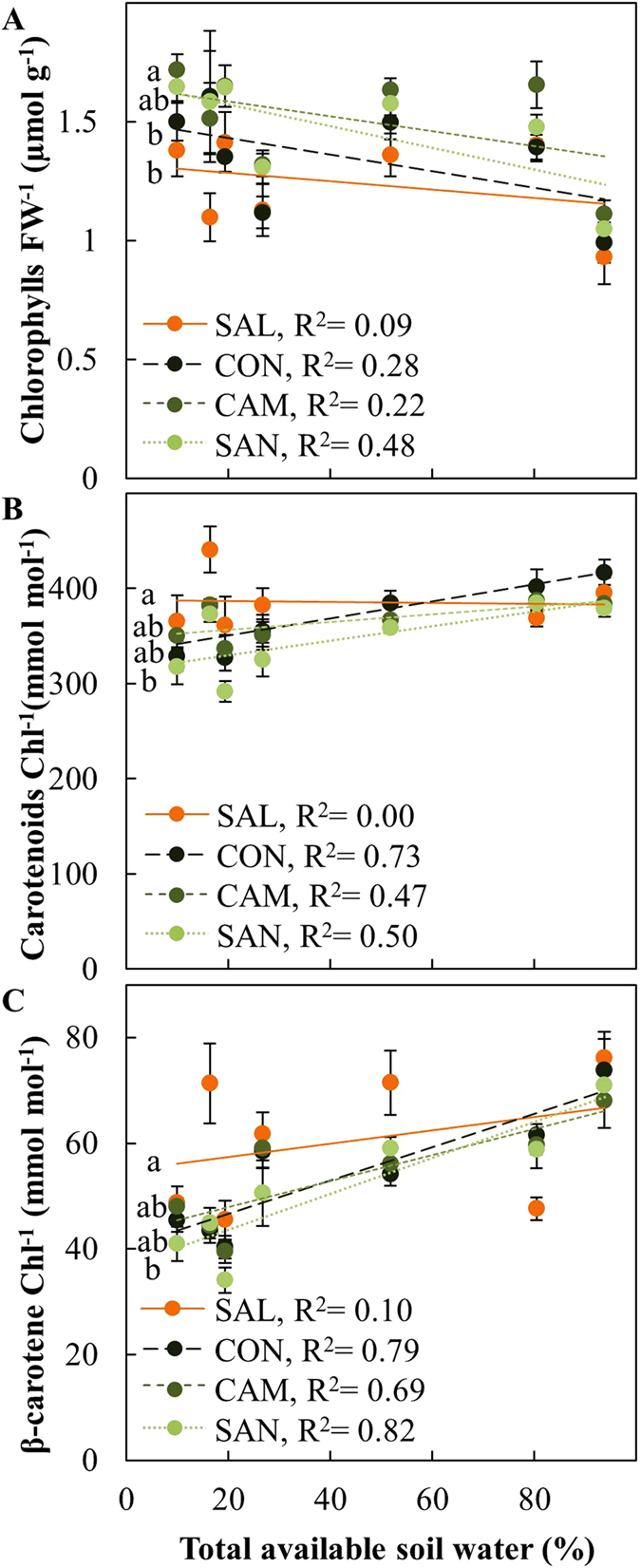
Photosynthetic pigment content of four Douglas-fir provenances in response to total available soil water. (**A**) total chlorophyll a and b per fresh weight (chlorophylls FW^−1^), (**B**) carotenoids per total chlorophyll (Carotenoids Chl^−1^), (**C**) β-carotene per total chlorophyll (β-carotene Chl^−1^) were measured in May and July of 2010 and 2011 at two field sites, Schluchsee and Wiesloch. Data was obtained from n = 5–6 samples of previous year needles (±SE). Data obtained from Schluchsee in May 2010 was excluded (see material and methods for further details). Significant differences between provenances (p < 0.05) are indicated by different letters.

Chlorophyll content was higher at the dry site Wiesloch compared to Schluchsee and thus was higher when TAW was low and showed significant variation between provenances (Fig. 6A). During all campaigns, SAL and CON showed the lowest chlorophyll content, whereas CAM showed the highest content and SAN intermediate values (Fig. S3A). Lower chlorophyll content under low TAW also affected the ratio of carotenoids per chlorophyll. This ratio was typically slightly lower as TAW decreased (Fig. 6B). In response to a decrease in TAW, the ratio of carotenoids per chlorophylls remained highest in SAL, followed by CON and CAM and lowest in SAN. However, it must be noted that the highest carotenoid content was observed under cold conditions early in the growing season in May 2010 in Schluchsee (Fig. S3C). The β-carotene content of needles also showed a decrease in response to soil water availability, which was less pronounced in SAL compared to the coastal provenances (Fig. 6C). Provenance-specific variation in β-carotene was especially pronounced under dry conditions in July 2010 in Wiesloch (Fig. S4A).

**Figure 7:**
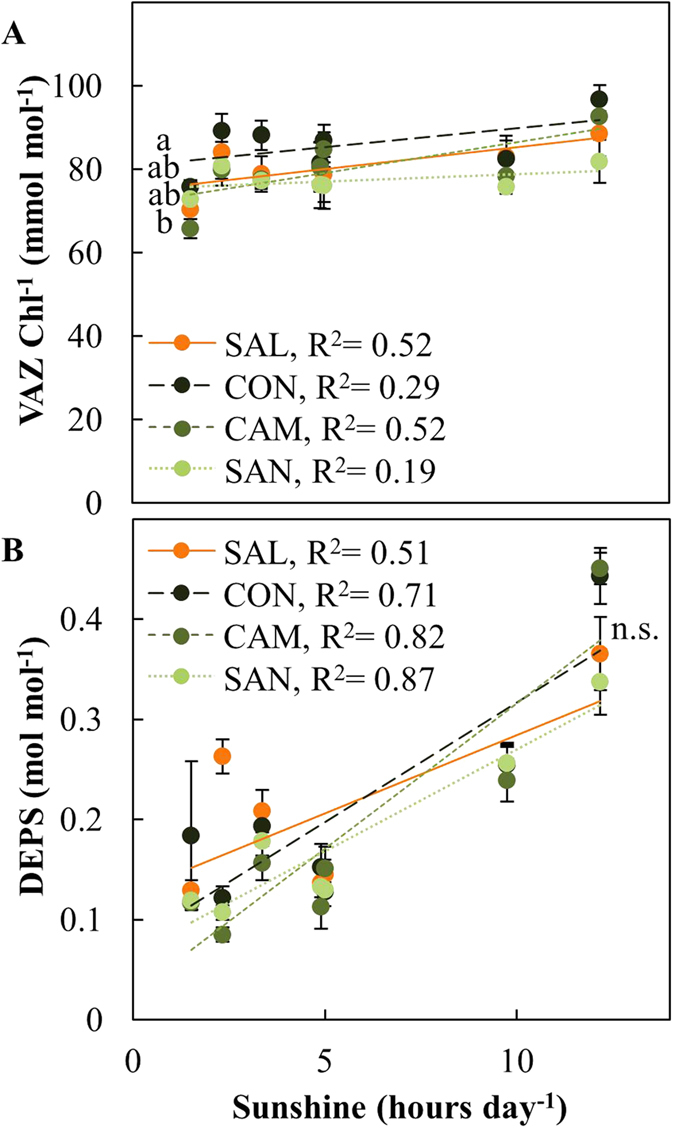
Photosynthetic pigment content of four Douglas-fir provenances in response to sunshine duration per day as proxy for solar radiation. (**A**) Xanthophyll cycle pigments per total chlorophyll (VAZ Chl^−1^), and (**B**) de-epoxidation status of the xanthophyll cycle pigments (DEPS) were measured in May and July of 2010 and 2011 at two field sites, Schluchsee and Wiesloch. Data was obtained from n = 5–6 samples of previous year needles (±SE). Data obtained from Schluchsee in May 2010 was excluded (see material and methods for further details). Significant differences between provenances (p < 0.05) are indicated by different letters.

The pool of xanthophyll cycle pigments (VAZ) increased in response to sunshine duration per day, which is a proxy for solar radiation (Fig. 7A). CON showed consistently highest VAZ-pools, followed by SAL, CAM and SAN. Highest VAZ-pools sizes under summer conditions were observed in July 2010 in Wiesloch when we observed 12 h of daily sunshine duration combined with lowest TAW. Here, VAZ-pools of all provenances were about 15–20% larger than in Schluchsee (Fig. S4B). Nonetheless, highest VAZ-pools in Schluchsee were not observed in response to water limitations and high light episodes, but during early season low temperature conditions in May 2010 (Fig. S4B). The de-epoxidation state (DEPS) of the VAZ-pool, which reflects the photoprotective conversion of violaxanthin into zeaxanthin and antheraxanthin, was strongly increased under sunny conditions, but we did not observe any variation among provenances (Fig. 7B). High VAZ-pool sizes observed under very sunny and dry conditions in July 2010 in Wiesloch were paralleled by a threefold increase in DEPS, (Fig. S4B).

**Figure 8:**
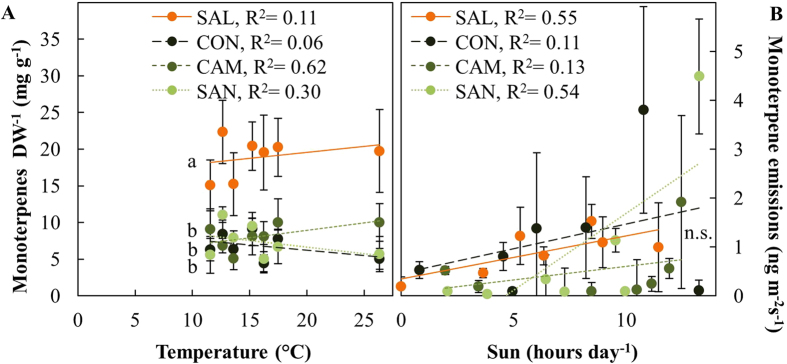
Monoterpene pools and emission of four Douglas-fir provenances in response to sunshine duration per day as a proxy for solar radiation. (**A**) monoterpene pool sizes (monoterpenes DW^−1^), and (**B**) monoterpenes emitted per needle area (monoterpene emissions) were measured in May and July of 2010 and 2011 at two field sites, Schluchsee and Wiesloch. Data was obtained from n = 5–6 samples of previous year needles (±SE). Monoterpene pools include L-α-bornyl acetate, camphene, 3-carene, (+)-4-carene, β-citronellal, β-citronellol, citronellyl acetate, geraniol acetate, isoprene, (−)-isopulegol, limonene, linalool, β-myrcene, nerolidol, ocimene, β-phellandrene, α-pinene, β-pinene, sabinene, α-terpinene, γ-terpinene, α-terpineol, L-4-terpineol and tricyclene. Data obtained from Schluchsee in May 2010 was excluded (see material and methods for further details). Significant differences between provenances (p < 0.05) are indicated by different letters.

The needle concentrations of stored monoterpenes did not significantly correlate with any environmental factor, and they were consistently higher in SAL than in any of the coastal provenances (Fig. 8A, Supplementary Fig. S5A). Monoterpene emission rates were highly variable among individual trees, but significantly correlated with sunshine duration per day (Fig. 8B, Supplementary Fig. S5B).

## Discussion

We assumed that Douglas-fir provenances vary in photosynthesis and isoprenoid-mediated photoprotective mechanisms in response to drought. Specifically, we expected that all provenances employ short-term photoprotective responses to drought, but reveal provenance-specific differences in long-term adjustments. Our results showed that provenances vary in photosynthetic gas exchange under intermediate to high TAW at the Schluchsee site (Figs 1 and 2). In contrast, at the Wiesloch site, photosynthesis in all provenances was strongly limited by generally low TAW. Under these predominantly drier soil conditions, provenances revealed variation in long-term adjustments of the composition of essential isoprenoids (Figs 2 and 3).

The observed good correlation of net CO_2_ assimilation (A) and stomatal conductance (g_s_) with TAW (Fig. 4A,B) is typical for conifers, which control water loss through transpiration by decreasing g_s_ in response to decreasing soil water availability^9^,^33^. Trees with an increased drought-tolerant manage water loss better and are thus able to maintain higher assimilation rates under drought^34^. Consequently, slightly higher A and g_s_ in SAL suggests increased drought tolerance of this interior provenance compared to the three coastal provenances. The effect of local adaptation on photosynthetic performance, as was previously shown e.g. for poplar^35^ and eucalyptus^36^, was furthermore revealed by provenance-specific variation in discrimination against stable carbon isotopes (Δ^13^C_WSOM_). Δ^13^C_WSOM_ is a time-integrated proxy of the intrinsic water use efficiency (IWUE) that exacerbates potential differences in A/g_s_^37^,^38^. Although the A/g_s_ ratio was rather invariable (Fig. 2C), a positive correlation between Δ^13^C_WSOM_ and TAW indicated enhanced IWUE^30^,^39^ in response to drought (Fig. 4C, Table 2). Lower Δ^13^C_WSOM_ in CON and SAN furthermore suggests enhanced IWUE in these two coastal provenances compared to SAL and CAM (Fig. 4C). Similar to the observations by Jansen *et al*.^30^ and Zhang *et al*.^40^ our data reveal intraspecific variation in IWUE with coastal provenances generally showing higher IWUE compared to to interior provenances.

Provenance-specific differences in photosynthetic gas exchange were not reflected by chlorophyll fluorescence parameters. For example, the optimum quantum yield of photosynthesis F_v_/F_m_, is typically a sensitive indicator for photoinhibition under high light stress and in in response to drought^41^. However, we only observed decreases in F_v_/F_m_ under extremely dry conditions in July 2010, when yield of PSII was also minimal (Supplementary Fig. S2A,B). High F_v_/F_m_ values in combination with a low yield of PSII (Fig. 5A,B) indicates that the photosynthetic apparatus remains intact during drought and the downregulation of yield of PSII reflects dynamic short-term response to mitigate acute excess energy and increased photoprotection against oxidative stress^19^. Since this coincided with enhanced non-photochemical quenching (NPQ) in response to decreased TAW (Fig. 5C) we likely observed photoprotective feedback response^42^ to decreases in A and g_s_ that occurred under dry conditions.

We had hypothesized, that provenance-specific differences in photosynthesis go along with differences in a suite of isoprenoid-mediated photoprotective mechanisms. We expected that provenances with low photosynthetic performance during drought, e.g. SAN and CAM, will show enhanced photoprotection by essential isoprenoids, including chlorophylls and carotenoids.

In contrast to our prediction, SAL showed low chlorophyll content and enhanced carotenoid-chlorophyll ratio (Fig. 6A,B, Table 2), which were previously attributed to enhanced photoprotection in various tree species including *Picea asperata, Pinus halepensis* and *Quercus pubescens*^43^,^44^,^45^. SAL also exhibited higher amounts of β-carotene compared to coastal provenances, but revealed high plasticity (Fig. 6C, Supplementary Fig. 4A). β-carotene pool sizes are affected by long-term adjustments of the photosynthetic apparatus to prevailing light conditions^13^,^23^ as well as by short-term oxidation reactions^21^,^22^. The decrease of β-carotene in the coastal provenances CON, CAM and SAN in response to low TAW thus suggests, that β-carotene biosynthesis was not sufficient to combat the oxidation of β-carotene caused by chemical scavenging of ROS^20^. Overall, the enhanced pools of essential isoprenoids in SAL suggests enhanced photoprotection of the photosynthetic apparatus, suggesting that essential isprenoids are important contributors to the overall higher assimilation rates of this provenance.

We also determined the involvement of the xanthophyll cycle, an ubiquitous photoprotective mechanism^2^,^13^,^19^ in the adjustment of the photoprotective capacity of Douglas-fir in response to drought. The long-term adjustment of the xanthophyll cycle pigment (VAZ) pool size in response to sunshine duration per day (used here as a proxy for global radiation^46^; Fig. 7A) provides evidence for an upregulation of the photoprotective capacity in response to abiotic stress conditions in Douglas-fir. Although differences in pool sizes among provenances seem to be small, with largest VAZ-pools in CON and smallest pools in SAN, the pattern was consistent and revealed significant provenance-specific variation (Table 2) and likely reflects local adaptation to contrasting habitats. Provenance-specific variation in essential isoprenoids was most pronounced under extremely dry, hot and sunny conditions in July 2010 at Wiesloch when the demand for photoprotection was highest throughout all our campaigns (Table 1, Supplementary Figs S3 and 4). During the same campaign at Wiesoch we also observed in all provenances a maximum in the de-epoxidation state of VAZ (DEPS, Fig. 7B), which is an indication of a short-term and instantaneous response to mitigate photo-oxidative stress induced by high light^47^,^48^.

Compared to carotenoids, which are essential and conserved in higher plants^49^, non-essential isoprenoids are highly variable across species^50^ and within species^51^,^52^. This variability was reflected by the observed provenance-specific differences in monoterpene pool sizes, which exceeded the observed variations in essential isoprenoids by far (Figs 3F and 8A).

Stored monoterpenes indicate long-term adjustments to environmental conditions^53^,^54^. In contrast, the emission of monoterpenes is instantaneously driven by prevailing environmental conditions to mitigate acute abiotic stress^26^,^27^,^55^. Emission of monoterpenes correlated with sunshine duration per day, but showed large variability among trees and campaigns, without provenance-specific differences (Figs 3G and 8B, Supplementary Fig. S5B). This is likely due to a high percentage of *de novo* biosynthesized monoterpenes which have been shown to constitute up to 58% of emitted monoterpenes in *Pinus sylvestris*^56^. Emitted monoterpenes represent a mix of stored (temperature dependent) and *de novo* synthesized (light and temperature dependent) monoterpenes^57^ which may consequently contribute to the lack of a relationship between pool sizes and rates of emission and large variation in emission rates (Fig. 8A,B).

Despite their possible function in mitigating photooxidative stress, the emission of monoterpenes implies a loss of previously fixed CO_2_. This loss in previously fixed CO_2_ can be substantial and for beech seedlings it was shown that it can even result in reduced growth^29^. Previously it was shown that interior provenances reveal reduced growth compared to coastal provenances^6^, an observations that is also confirmed for the provenances studied here and at our field sites^58^, Based on our observations, increases in the allocation of previously fixed CO_2_ to the biosynthesis of monoterpenes for storage and emission in the provenance SAL might therefore affect its growth performance compared to coastal provenances.

In conclusion, our results reveal provenance-specific variation in g_s_ and IWUE, indicating local adaptation to habitats with contrasting soil water availability. All provenances shared similar short-term photoprotective responses when photosynthetic CO_2_ uptake was decreased due to low TAW. These photoprotective responses involved higher rates of NPQ, as well as increased de-epoxidation of the xanthophyll cycle pigments and enhanced emission of volatile isoprenoids under high light conditions. In contrast to these provenance-wide responses, we also observed provenance-specific differences in long-term adjustments represented by differences in the pool sizes of xanthophyll cycle pigments, β-carotene and stored monoterpenes in response to drought and high light. Provenance-specific variation in essential and non-essential isoprenoids therefore seem to reflect local adaptation in isoprenoid-mediated photoprotective mechanisms. Most importantly, we did not observe the highest photoprotection in the provenances, that exhibit lower assimilation rates, as suggested by our hypothesis, but rather in the provenances, which were able to maintain higher assimilation rates. Therefore, isoprenoid-mediated photoprotective mechanisms seem to contribute to better adaptation of species to warmer and drier climate and to serve as an important trait to enhance forest ecosystem resilience.

## Materials and Methods

### Field sites and plant material

We compared trees at two field sites in south-western Germany which are part of an international Douglas-fir (*Pseudotsuga menziesii*) provenance trial established in 1958^59^. “Schluchsee” is located in the southern Black Forest (1050 m a.s.l) and represents a moderately cool, humid climate with 1345 mm mean annual precipitation and a mean annual temperature of 6.1 °C. “Wiesloch” is located in the Rhine valley (105 m a.s.l.) and is characterized by warmer and drier climatic conditions with 9.9 °C mean annual temperature and 660 mm mean annual precipitation^60^. Meteorological data for 2010 and 2011 were obtained from nearby weather stations (Table 3). This included air temperature, precipitation and daily sunshine duration, measured as the sum of hours when irradiance exceeds 120 W m^−2 ^61. Sunshine duration has been shown to be a suitable proxy for solar irradiance due to the linear relationship between both parameters^46^,^62^. The 1961–1990 climate reference (Deutscher Wetterdienst, DWD) was used to characterize the long term climate at the sites. Soil water availability was calculated using the forest hydrological water budget model WBS3. The model estimates daily total available soil water (TAW) using temperature, precipitation, latitude, soil type, plant cover, slope, and slope aspect^63^. At both sites, we compared the interior provenance Salmon Arm (SAL) (*var. glauca*, originating from a dry habitat in British Columbia) and three coastal provenances Conrad Creek (CON), Cameron Lake (CAM), and Santiam River (SAN) (*var. menziesii*, all originating from humid habitats, see Table 4).

### Measurement campaigns

Field work was conducted in May and July of 2010 and 2011 at both sites and included a total of eight measurement campaigns. Each campaign lasted two weeks. Because of the higher elevation of the Schluchsee site, the growing season begins two to three weeks later than at the Wiesloch site. Field measurements in Wiesloch were therefore carried out prior to the measurements in Schluchsee. Phenology of bud development was assessed in eight samples per provenance using an index with five classes according to Bailey and Harrington^64^ (Supplementary Table S1). Gas exchange and chlorophyll fluorescence measurements were conducted in the sun-exposed crown of the trees at heights between 24 to 29 meters in 5–6 trees per provenance using a platform on a hydraulic lift. At the end of each campaign, needle material of the sun-exposed crown of 6 trees per provenance was sampled nearly simultaneously between 12 pm and 2 pm using shotguns or slingshots. Previous year needles were sampled from the twigs, immediately frozen in liquid nitrogen and stored at −80 °C. In 2011, pre-dawn and midday twig water potential was determined for 4 trees one day before or after the needle sampling day to assess xylem water tension. Water potential from freshly cut two-year-old twigs was determined between 6:00 and 8:00 am and 1:00 and 3:00 pm using a pressure chamber (Model 3015G4, Soil moisture Equipment Corp., Santa Barbara, CA, USA) according to Scholander *et al*.^65^.

### Photosynthesis measurements

Chlorophyll fluorescence and gas exchange were measured in previous year needles on branches within the sun-exposed crown using a LI-COR 6400 XT with an integrated 6400–40 leaf chamber fluorometer (LI-COR Biosciences, Lincoln, NE, USA). To ensure that all measurements are comparable despite variation in environmental light conditions, measurements followed a standardised protocol and were carried out between 10:00 am and 6:00 pm using the internal light source of the leaf chamber fluorometer. Prior to starting the gas exchange measurements, needles on an intact twig were dark-adapted using the LI-COR dark adapting clip kit. After 25 min, about 10–15 needles forming a flat area were enclosed by the cuvette. Measurement conditions in the closed cuvette were set to a flow rate of 400 ml min^−1^, 25 °C block temperature, 35% relative humidity, and a CO_2_ concentration of 400 ppm. Maximal and minimal fluorescence of the dark-adapted sample (F_o_ and F_m_) as well as dark respiration (R) were then assessed. Subsequently, gas exchange and chlorophyll fluorescence were measured at 1000 μmol photons m^−2^ s^−1^ light intensity after steady state of photosynthetic CO_2_ gas exchange was achieved, typically after 10–12 min. After the measurement, the light exposed needle surface area was determined using WinSeedle software and scanner (Regents Instruments Inc., Québec, Canada). The rate of photosynthetic gas exchange was expressed per projected needle area exposed to the light. Intrinsic water-use efficiency (IWUE) was calculated as the ratio of net CO_2_ assimilation rate to stomatal conductance (A/g_s_). The maximum quantum yield of dark-adapted needles was calculated as the ratio of variable to maximum chlorophyll fluorescence (F_v_/F_m_ = (F_m_ − F_o_)/F_m_), yield was calculated from light-adapted needles as (Φ_PSII_ = (F_m_’ - F_t_)/F_m_’), and non-photochemical quenching was calculated as NPQ = (F_m_ − F_m_’)/F_m_’, following Maxwell & Johnson^66^. Furthermore, the external quantum sensor of the LI-COR 6400XT was used to record photosynthetic photon flux density (PPFD). Since measurements were strongly affected by angle towards the sun and shading, maximum values per day were averaged per campaign (Table 1).

### Analysis of photosynthetic pigments

Pigments were extracted using 98% methanol buffered with 0.5 M ammonium acetate and analysed by HPLC-DAD according to a protocol modified from Ensminger *et al*.^14^. An Agilent high performance liquid chromatography (HPLC) system (Böblingen, Germany) with a quaternary pump (model 1260), autosampler (model 1260, set to 4 °C), column oven (model 1260, set to 25 °C), and photodiode array detector (model 1290, recording absorption at 290 nm, 450 nm and 656 nm wavelength) was used for reverse-phase chromatography using a C_30_-column (5 μm, 250*4.6 mm; YMC Inc., Wilmington, NC, USA). Three solvents (A: 100% methanol, B: 60% methanol buffered with 20 mM ammonium acetate, C: 100% methyl-tert-butyl-ether) were used to run a gradient starting with 40% A and 60% B. Solvent B was gradually replaced by solvent A to a minimum of 5% B; afterwards solvent A was gradually replaced by solvent C until the solvent mixture consisted of 45% A, 5% B and 50% C. Peaks were quantified using standards for chlorophyll a, chlorophyll b and β-carotene from Sigma Aldrich (Oakville, ON, Canada). Standards for violaxanthin, antheraxanthin and zeaxanthin were obtained from DHI Lab products (Hørsholm, Denmark). ChemStation B.04.03 software (Agilent Technologies, Böblingen, Germany) was used for peak integration.

### Analysis of monoterpene pools and monoterpene emissions

The 8-cm^2^-cuvette of a portable gas exchange analyser (GFS-3000, Walz, Effeltrich, Germany) was closed around previous-year-needles of an intact sun-exposed twig. Care was taken to prevent needle injury causing emission of stored monoterpenes. The observed monoterpene emission was similar to that observed in other studies on Douglas-fir^67^, and the composition of emitted monoterpenes was considerably different from that of stored monoterpenes (data not shown), clearly indicating that emissions from injured needles were negligible in our study. The cuvette was flushed with 650 ml min^−1^ of compressed air (Air Liquid, Ludwigshafen, Germany) at 35% relative humidity, 400 ppm CO_2_ concentration, 30 °C leaf temperature and 1000 μmol m^−2^ s^−1^ light intensity. After equilibration of photosynthesis to these conditions, air was drawn from the cuvette through an air sampling tube packed with 20 mg Tenax TA 60/80 and 30 mg Carbotrap B 20/40 (Supelco, Bellafonte, PA, USA) for 40 min at a flow rate of 150 ml min^−1^ using an air sampling pump (Analyt-MTC, Müllheim, Germany). Air sampling tubes were then stored in glass vials at 4 °C. The area of the needles enclosed in the cuvette was determined as described above. Monoterpene emission rates were calculated per leaf area and over time and corrected by subtracting zero references, which were taken frequently using an empty cuvette to correct for background contaminations.

Monoterpenes stored in needles were extracted in 500 μl methanol per 25 mg frozen ground needle material for 20 min while the suspension was agitated and kept at 30 °C. Extracted monoterpenes were diluted and quantitatively bound to polydimethylsiloxane (PDMS) coated Twisters^®^ (Gerstel, Mülheim, Germany) by stirring them at 1400 rpm for 60 min at 30 °C. Twisters were dried with a lint free paper tissue and placed into glass tubes.

Analysis of emitted and stored monoterpenes was performed by gas chromatography-electron impact mass spectrometry (GC-EI/MS) according to Ghirardo *et al*.^56^. Peaks were identified and quantified with external standards and by comparison of the de-convoluted fragmentation spectra with the NIST database using the AMDIS software (National Institute of Standards and Technology (NIST), Gaithersburg, MD, USA). Needle monoterpene concentrations were calculated per gram needle dry weight.

### ^13^C isotope discrimination measurements

Following Gessler *et al*.^68^ and Ruehr *et al*.^69^, the isotopic composition (δ^13^C_WSOM_) of the water-soluble organic matter (WSOM) fraction of the needles (mainly sugars, but also some amino acids and organic acids) was analysed with an elemental analyzer coupled to an isotope ratio mass spectrometer (Delta V Advantage, ThermoFisher, Bremen, Germany). Carbon isotopic values were expressed in δ notation relative to the Vienna Pee Dee Belemnite (VPDB) standard. The precision for measurements as determined by repeated measurements of standards (N = 10) was better than 0.1‰. δ^13^C_WSOM_ values were corrected for the effect of reduced O_2_ partial pressure at higher elevation assuming an increase in δ^13^C of 0.22‰ per 100 m^63^,^70^. From δ^13^C_WSOM_ and tropospheric CO_2_ (δ^13^C_atm_), we calculated the photosynthetic carbon stable isotope discrimination (Δ^13^C_WSOM_).

δ^13^C_atm_ was based on averaged monthly data from long-term measurements at the station Schauinsland (Freiburg, Germany) between the years 1977–1996^71^, and corrected for a mean decrease in δ^13^C_atm_ by 0.017‰ yr^−1^ and for a methodology based offset of 0.2‰ as reported by Levin & Kromer^71^. Δ^13^C_WSOM_ is a proxy for IWUE^72^. Δ^13^C_WSOM_ of leaves and needles is known to integrate IWUE over a period of hours to days^38^,^73^.

### Statistics

All statistical tests were performed using R 3.0.3^74^. The effect of site (environment effect) and provenance (genotype effect) and the interaction thereof on physiological parameters for photosynthetic performance and stored and emitted isoprenoids across all sampling time points were assessed using two-way ANOVA including time of campaign as random effect (function *aov*, see Table 2). Homogeneity of variance and normality of distribution were tested by Levene’s test and Shapiro-Wilk-Test, respectively (function *levene* from the library *car* and *shapiro.test*). Differences between provenances across field sites and across time points (see Figs 2 and 3) were estimated using a corresponding linear mixed-effect model (Site x Provenance, time as random effect; function *lmer*, package *lme4*, Bates *et al*.^75^) followed by the determination of least-squares means (function *lsmeans*) between provenances for all physiological parameters where *provenance* was significant, using the R package *lsmeans*^76^. Pairwise differences between provenances were estimated and the significance of the contrasts was assessed using Tukey’s multiple comparison test (Figs 2 and 3).

The correlation between physiological parameters and the three environmental factors *TAW* (total available soil water), *Sun* (sunshine duration), and *Temperature* (mean daily temperature) on the day of measurement or sampling, respectively, was performed using Pearson’s product-moment correlation coefficient (function *cor*, method *pearson*). When interaction with any environmental parameter was significant, physiological parameters were plotted against the environmental factor, that showed the highest correlation. To enhance readability of the graphs, data was averaged per campaign. Differences among provenances are displayed by linear regression and significances were estimated using the corresponding linear model followed by Tukey’s multiple comparison test of least-squares means.

For all abovementioned statistics, data obtained in May 2010 in Schluchsee were omitted due to the drastically different environmental conditions at the beginning of the growing season. The start of the growing season can be marked by the first day when mean daily temperature consistently exceeds 5 °C^77^. In Schluchsee in May 2010 this threshold was exceeded only two days before our measuring campaign began. For all other May campaigns, the growing season had started already 30–50 days earlier. The late start of the growing season in May 2010 in Schluchsee was also revealed by the phenology data for bud development (Supplementary Table S1), and in Supplementary Figures S1–S5, where the physiological data are presented by campaign and field site. For Supplementary Figures S1–S5, the differences between provenances within sites and at each sampling time point was determined by a separate one-way ANOVA (function *aov*), followed by Tukey’s post hoc test (function *TukeyHSD*).

Differences in bud development between campaigns and provenances as shown in Supplementary Table S1 were estimated using Kruskal-Wallis-Rank-Sum-Test (function *kruskal.test*).

## Additional Information

**How to cite this article:** Junker, L. V. *et al*. Variation in short-term and long-term responses of photosynthesis and isoprenoid-mediated photoprotection to soil water availability in four Douglas-fir provenances. *Sci. Rep.* **7**, 40145; doi: 10.1038/srep40145 (2017).

**Publisher's note:** Springer Nature remains neutral with regard to jurisdictional claims in published maps and institutional affiliations.

## Supplementary Material

Supplementary Data

## Figures and Tables

**Table 1:** Weather conditions and twig water potential during measurement campaigns.

	Schluchsee								Wiesloch							
	2010				2011				2010				2011			
	May		July		May		July		May		July		May		July	
Precipitation, cumulated over 14 days (mm)	100.2	±41.8	71.4	±23.2	24.0	±7.3	96.8	±25.7	29.2	±15.4	5.8	±6.9	4.3	±1.0	36.7	±11.0
Temperature (°C)	10.9	±4.3	14.7	±3.1	13.0	±2.5	11.6	±1.6	10.4	±2.0	24.8	±2.6	16.3	±4.2	19.0	±2.6
Sun (h)	6.4	±4.8	6.6	±4.8	8.2	±4.1	4.0	±1.9	1.8	±1.8	11.5	±2.9	10.3	±3.6	5.4	±3.5
TAW (%)	89.6	±6.5	80.1	±3.8	33.2	±3.0	96.7	±2.7	45.7	±6.5	9.6	±3.2	10.6	±1.1	20.0	±7.2
PPFD (μmol m^−2^ s^−1^)	49	±22	1198	±397	894	±545	978	±688	449	±245	577	±468	847	±619	276	±132
Pre-dawn twig water potential (MPa)	NA		NA		−0.68	±0.10	−0.53	±0.13	NA		NA		−0.85	±0.11	−0.64	±0.11
Midday twig water potential (MPa)	NA		NA		−1.50	±0.23	−1.61	±0.26	NA		NA		−1.69	±0.18	−2.05	±0.33

14-day-cumulative precipitation, mean daily temperature, sunshine duration (Sun), total available soil water (TAW) and photosynthetic photon flux density (PPFD) were averaged over the duration of field campaign (±SD). Twig water potential of Douglas-fir (*Pseudotsuga menziesii*) was assessed at the end of each campaign in 2011 in n = 12 trees, equally distributed between provenance (±SD; no provenance-specific differences were detected). NA = Not assessed.

**Table 2:** Effect of *Provenance* and *Site* on photosynthetic gas exchange, isotope discrimination, chlorophyll fluorescence, and isoprenoids assessed by two-way ANOVA, as well as correlation between these physiological parameters to environmental conditions revealed by Pearson-correlation.

Parameter	ANOVA			Pearson-Correlation		
	*Provenance*	*Site*	*Prov:Site*	*TAW*	*Temperature*	*Sun*
A	**0.02**	**0.00**	**0.04**	**0.70**	**−0.42**	**−0.52**
g_s_	**0.04**	**0.00**	**0.00**	**0.73**	**−0.36**	**−0.48**
IWUE	0.07	0.49	0.07	0.04	−0.05	−0.17
R	0.54	0.09	0.19	−0.07	−0.08	−0.13
Δ^13^C_WSOM_	**0.00**	**0.00**	**0.01**	**0.62**	**−0.30**	**−0.22**
F_v_/F_m_	0.13	**0.00**	0.63	**−0.28**	**−0.26**	−0.02
Φ_PSII_	0.15	0.69	**0.02**	0.01	**−0.27**	**−0.25**
NPQ	0.73	**0.00**	0.96	**−0.54**	0.04	**0.22**
Chl a + b	**0.00**	**0.00**	0.40	**−0.33**	0.05	−0.08
Carotenoids	**0.00**	**0.00**	0.10	**0.35**	−0.05	−0.01
β-carotene	**0.00**	**0.00**	**0.00**	**0.56**	**−0.27**	**−0.16**
VAZ	**0.00**	0.24	0.93	−0.07	**0.33**	**0.29**
DEPS	0.19	0.27	0.32	**−0.21**	**0.65**	**0.71**
Monoterpenes	**0.00**	**0.04**	**0.00**	0.03	−0.02	−0.04
Emitted monoterpenes	0.31	0.07	0.27	−0.17	0.07	**0.29**

Section ANOVA shows the p-values from two-way ANOVA between *Provenance* and *Site* including interactions (*Prov:Site*). Section Pearson-Correlation shows the correlation coefficients of the Pearson’s product-moment correlation test for each physiological parameter to the three environmental variables total available soil water (*TAW*), mean daily temperature (*Temp*) and sunshine duration per day as proxy for solar radiation (*Sun*). Significance with p < 0.05 is indicated by bolded numbers. A = assimilation rate, g_s_ = stomatal conductance, IWUE = Intrinsic water-use efficiency, R = dark respiration, Δ^13^C_WSOM_ = discrimination against ^13^C in water soluble organic matter, F_v_/F_m_ = maximu_m_ quantum yield of dark-adapted needles, Φ_PSII_ = yield, NPQ = non-photochemical quenching, Chl a + b = total chlorophyll per fresh weight, Carotenoids = total carotenoids per total chlorophyll, VAZ = xanthophyll cycle pigments per total chlorophyll, DEPS = de-epoxidation status of the xanthophyll cycle pigments, monoterpenes = total stored volatile isoprenoids per dry weight, emitted monoterpenes = total monoterpene emissions.

**Table 3:** Climatic and geographical details of the field sites (elevation and mean annual parameters taken from Kenk & Ehring^60^).

Field site	Schluchsee	Wiesloch
Coordinates	N47°50′31′′, E8°6′54′′	N49°16′41′′, E8°34′37′′
Elevation	1050 m	105 m
Mean annual temperature	6.1 °C	9.9 °C
Mean annual precipitation	1345 mm	660 mm
Location of weather station	Schluchsee, 6 km distance N47°49′16′′, E8°11′08′′ (elevation 992 m), privately operated	Waghäusel-Kirrlach, 4 km distance N49°15′0′′, E8°32′24′′ operated by Deutscher Wetterdienst (DWD)

**Table 4:** Climatic and geographical details of the origin of the four Douglas-fir (*Pseudotsuga menziesii*) provenances used in the present experiments^60^.

Provenance	Elevation	Mean annual precipitation	Mean annual temperature
Salmon Arm (SAL)	650 m	500 mm	7.8 °C
Conrad Creek (CON)	280 m	2300 mm	9.5 °C
Cameron Lake (CAM)	210 m	1475 mm	10.0 °C
Santiam River (SAN)	800 m	1780 mm	9.5 °C
