# Supplementary material for: Variation in short-term and long-term responses of photosynthesis and isoprenoid-mediated photoprotection to soil water availability in four Douglas-fir provenances
Source: Sci Rep. 2017 Jan 10;7:40145. doi: 10.1038/srep40145 (PMC5223217; doi:10.1038/srep40145)

SUPPLEMENTARY DATA

Variation in short-term and long-term responses of photosynthesis and isoprenoid-mediated photoprotection to soil water availability in four Douglas-fir provenances

Laura Verena Junker, Anita Kleiber, Kirstin Jansen, Henning Wildhagen, Moritz Hess, Zachary Kayler, Bernd Kammerer, Jörg-Peter Schnitzler, Jürgen Kreuzwieser, Arthur Gessler, Ingo Ensminger

Table S1: Bud development of Douglas-fir provenances revealing differences in phenology during spring measurement campaigns. Bud development of four Douglas-fir provenances at sampling day in May differed significantly between field sites in 2010 (p<0.001) and between years 2010 and 2011 (p<0.001). Provenances only differ in May 2010 in Wiesloch (p=0.004). Bud development was categorized according to Bailey and Harrington [56] as mean of n=6-8 sampled twigs (±standard error) with 0 = dormant winter bud, 0.5 = slightly swollen and elongated bud with lighter tip, 1 = swollen buds of overall lighter colour with green tips, 1.5 = extremely swollen buds with visible green tissue and 2 = fully ruptured buds with protruding needles. Kruskal-Wallis analysis of variance was used to reveal significant differences between campaigns as indicated by letters, but did not detect differences between provenances.

	**Schluchsee**		**Wiesloch**	
	**2010[a]**	**2011[b]**	**2010[c]**	**2011[bd]**
**Salmon Arm**	0.88±0.13	2.00±0.00	1.81±0.19	2.00±0.00
**Conrad Creek**	0.69±0.09	2.00±0.00	0.69±0.09	1.88±0.08
**Cameron Lake**	0.75±0.09	1.94±0.06	1.63±0.21	1.94±0.06
**Santiam River**	0.69±0.09	1.94±0.06	1.19±0.21	2.00±0.00

Fig. S1: Photosynthetic gas exchange parameters of four Douglas-fir provenances. A) assimilation rates (A), B) stomatal conductance (g_s), C) intrinsic water-use efficiency (IWUE), D) carbon stable isotope discrimination ($\Delta^{13}C_{WSOM}$), and E) dark respiration rate (R_d) were measured in May and July of 2010 and 2011 at two field sites, Schluchsee and Wiesloch. Data of A, g_s, IWUE and R_d show means of n = 5-6 measurements (±SE) at a light intensity of 1000 µmol m^{-2} s^{-1}, data of $\Delta^{13}C_{WSOM}$ was obtained from n = 5-6 samples of previous year needles (±SE). Significant differences between provenances per campaign ($p < 0.05$ using one-way ANOVA followed by Tukey's HSD test) are indicated by different letters. Blue and yellow bars above figure show average total available soil water (TAW) and average sunshine duration (Sun) during measurement campaigns, respectively.

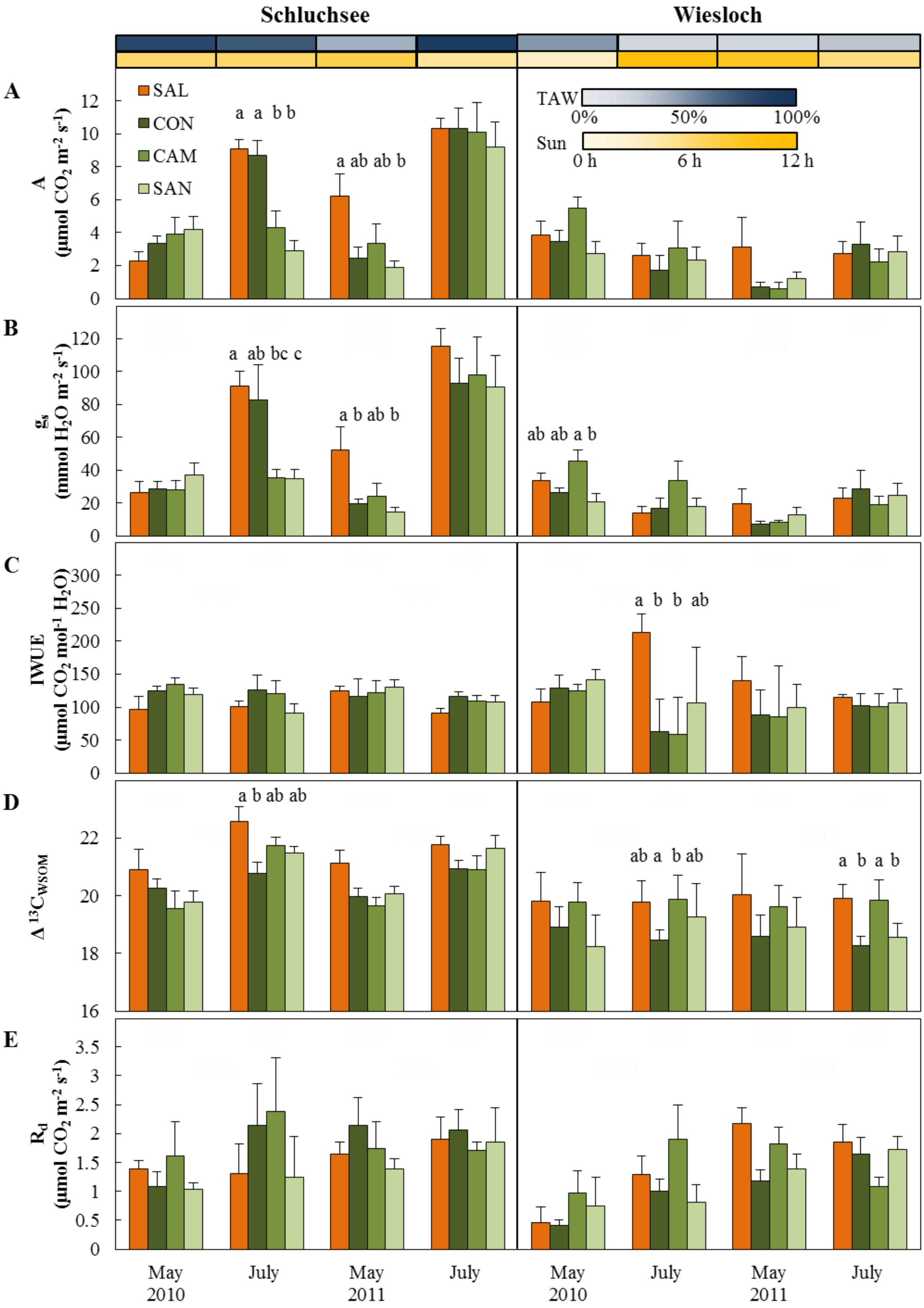

Schluchsee
Wiesloch
A
SAL
CON
CAM
SAN
TAW
0%
50%
100%
Sun
0 h
6 h
12 h
A
(μmol CO2 m-2 s-1)
a a b b
a ab ab b
B
gs
(mmol H2O m-2 s-1)
a ab bc c
a b ab b
ab ab a b
C
IWUE
(μmol CO2 mol-1 H2O)
a b b ab
D
Δ 13CWSOM
a b ab ab
ab a b ab
a b a b
E
Rd
(μmol CO2 m-2 s-1)
May 2010
July
May 2011
July
May 2010
July
May 2011
July

Fig. S2: Chlorophyll fluorescence parameters of four Douglas-fir provenances. A) maximum quantum yield of dark-adapted needles (F_v/F_m), B) effective quantum yield of light-adapted needles (ϕ_{PSII}), and C) non-photochemical quenching (NPQ) were measured in May and July of 2010 and 2011 at two field sites, Schluchsee and Wiesloch. Data of show means of n = 5-6 measurements (±SE) at a light intensity of 1000 µmol m^{-2} s^{-1}. Significant differences between provenances per campaign ($p < 0.05$ using one-way ANOVA followed by Tukey's HSD test) are indicated by different letters. Blue and yellow bars above figure show average total available soil water (TAW) and average sunshine duration (Sun) during measurement campaigns, respectively.

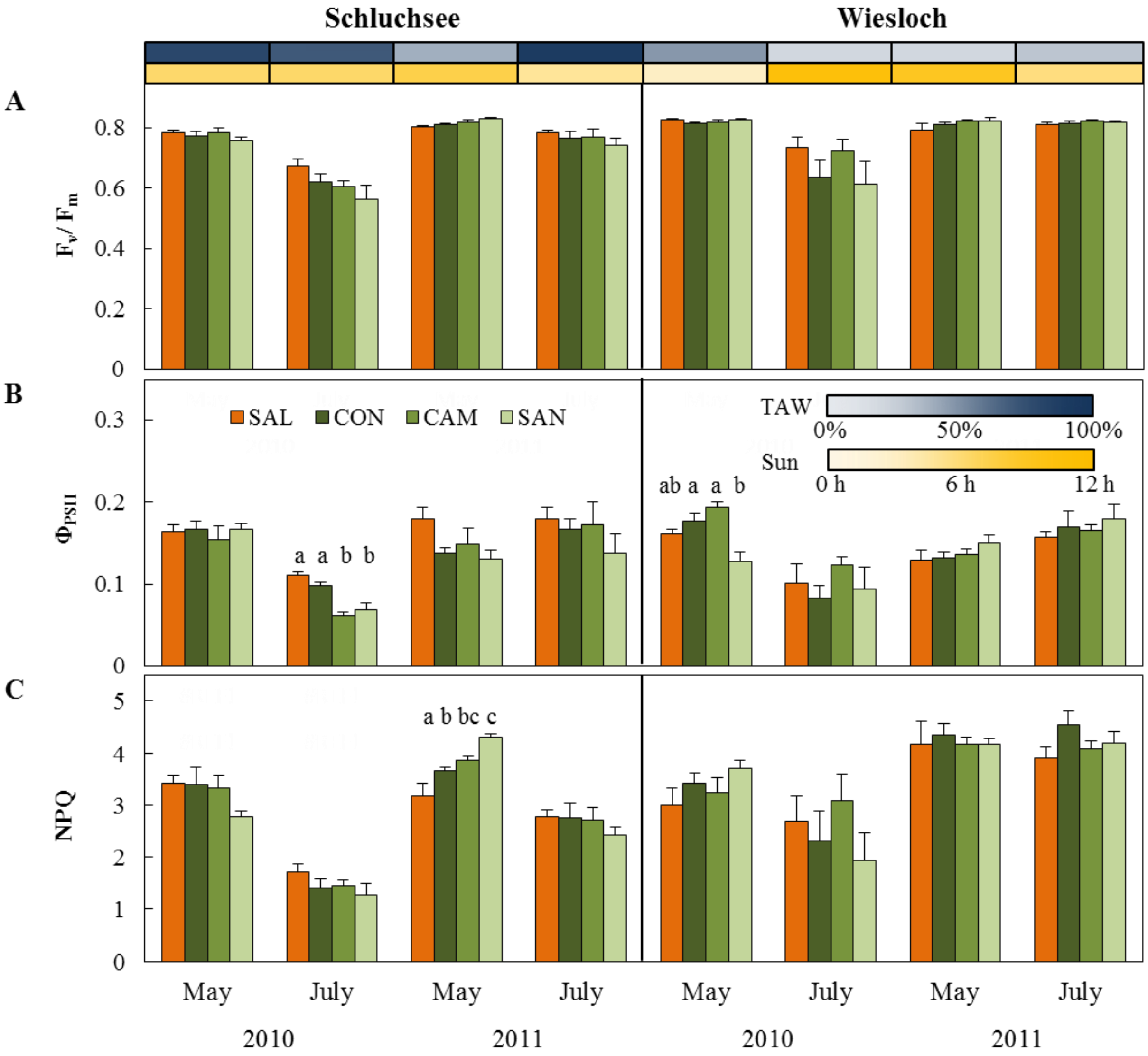

Fig. S3: Photosynthetic pigments of four Douglas-fir provenances. A) total chlorophyll a and b per fresh weight (chlorophylls FW^{-1}), B) chlorophyll a/b ratio (Chl a Chl b^{-1}), C) carotenoids per total chlorophyll (Carotenoids Chl^{-1}) were measured in May and July of 2010 and 2011 at two field sites, Schluchsee and Wiesloch. Data show means of n = 5-6 samples of previous year needles (±SE). Significant differences between provenances per campaign ($p < 0.05$ using one-way ANOVA followed by Tukey's HSD test) are indicated by different letters. Blue and yellow bars above figure show average total available soil water (TAW) and average sunshine duration (Sun) during measurement campaigns, respectively.

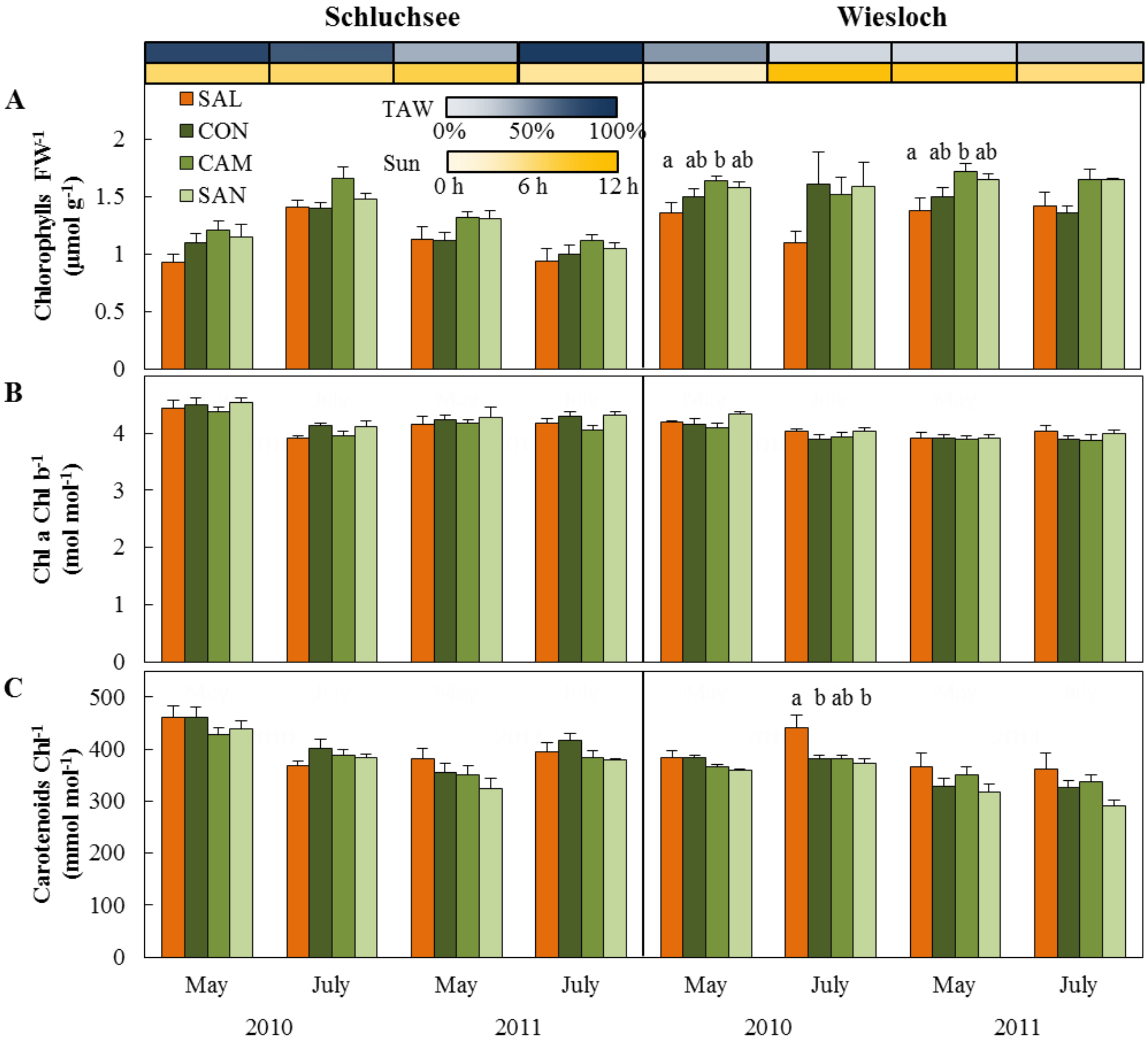

Fig. S4: Photosynthetic pigments of four Douglas-fir provenances. A) β-carotene per total chlorophyll (β-carotene Chl^{-1}), B) xanthophyll cycle pigments per total chlorophyll (VAZ Chl^{-1}), and C) de-epoxidation status of the xanthophyll cycle pigments (DEPS) were measured in May and July of 2010 and 2011 at two field sites, Schluchsee and Wiesloch. Data show means of n = 5-6 samples of previous year needles (±SE). Significant differences between provenances per campaign ($p < 0.05$ using one-way ANOVA followed by Tukey's HSD test) are indicated by different letters. Blue and yellow bars above figure show average total available soil water (TAW) and average sunshine duration (Sun) during measurement campaigns, respectively.

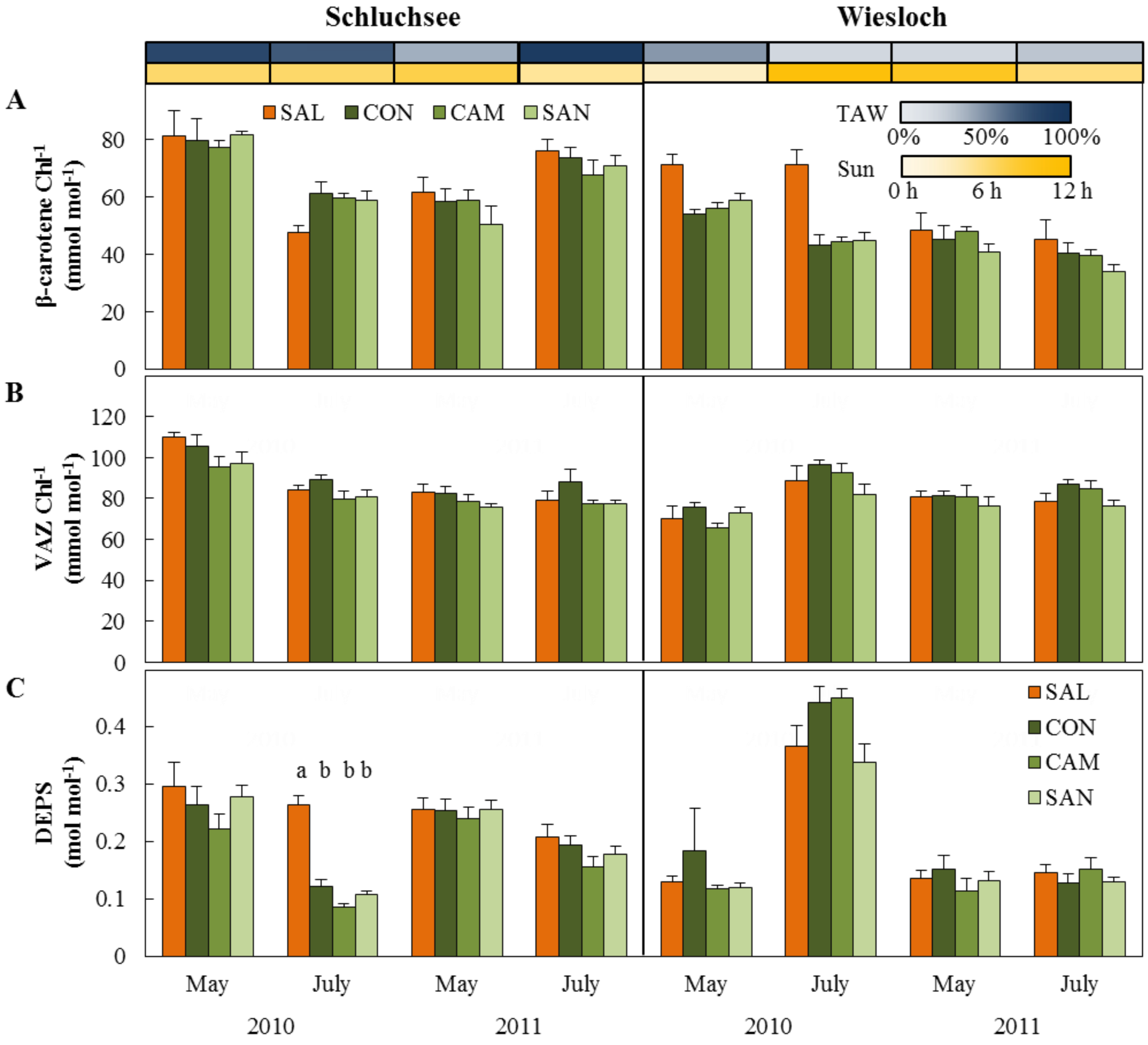

Fig. S5: Monoterpene pools and emission of four Douglas-fir provenances. A) monoterpene pool sizes (monoterpenes DW^{-1}), and B) monoterpenes emitted per needle area (monoterpene emissions) were measured in May and July of 2010 and 2011 at two field sites, Schluchsee and Wiesloch. Isoprenoid pool sizes were measured. Volatile isoprenoid pools were measured in n = 5-6 samples of previous year needles (±SE). Volatile isoprenoid emission was measured at 30 °C and 1200 µmol m^{-2} s^{-1} light intensity for n = 5-6 previous year needles (±SE). Significant differences ($p < 0.05$ using one-way ANOVA followed by Tukey's HSD test) are indicated by different letters. Upper (blue) and lower (yellow) bars above figure show average total available soil water (TAW) and average sunshine duration (Sun) during measurement campaigns, respectively.

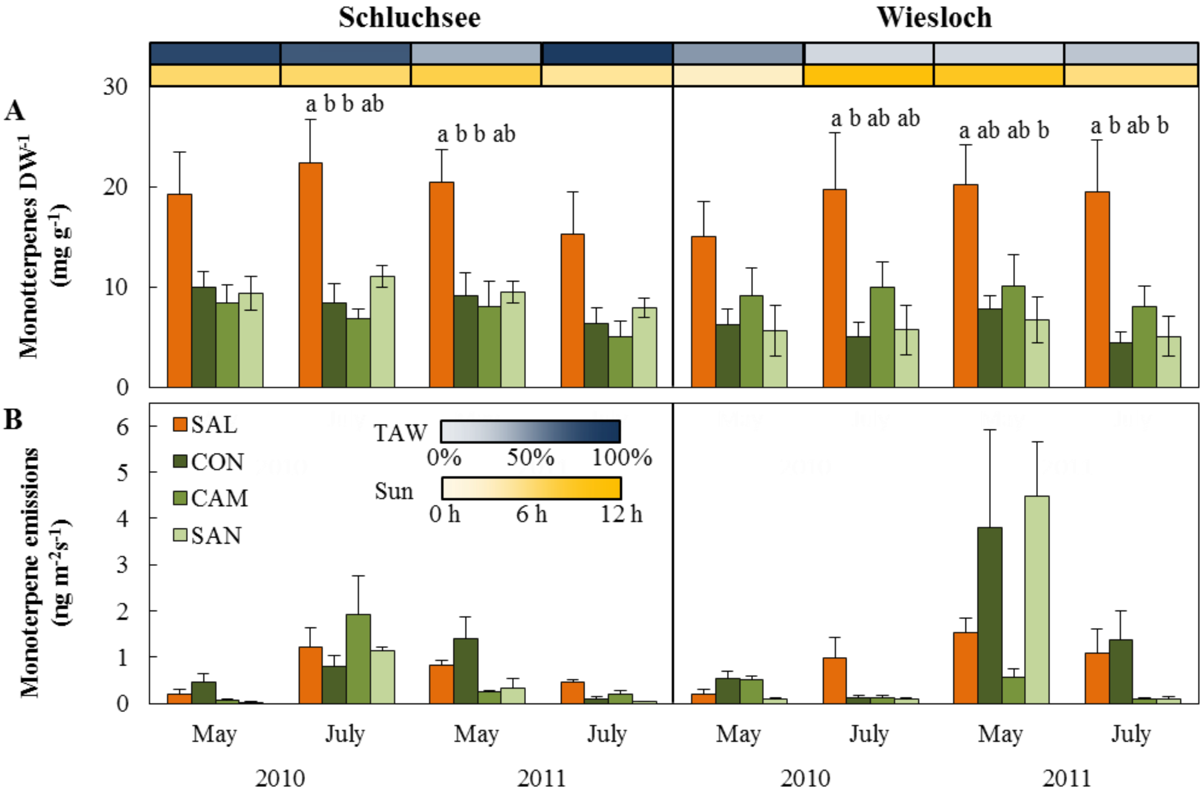

Supplement: Supplementary Data [file srep40145-s1.pdf]
